# Item response models for the longitudinal analysis of health-related quality of life in cancer clinical trials

**DOI:** 10.1186/s12874-017-0410-9

**Published:** 2017-09-26

**Authors:** Antoine Barbieri, Jean Peyhardi, Thierry Conroy, Sophie Gourgou, Christian Lavergne, Caroline Mollevi

**Affiliations:** 1 Biometrics Unit, Institut du Cancer Montpellier, 208 Avenue des Apothicaires, Montpellier, 34298 France; 2 Université de Montpellier, Place Eugène Bataillon, Montpellier, 34090 France; 3 Institut Montpelliérain Alexander Grothendieck, Montpellier, France; 4 Institut de génomique fonctionnelle, Montpellier, France; 5 French National Platform Quality of Life and Cancer, Nancy, France; 6 Institut de Cancérologie de Lorraine, Nancy, France; 7 University Paul-Valéry Montpellier 3, Montpellier, France; 8 Institut de Recherche en Cancérologie de Montpellier (IRCM) - Inserm U1194, Montpellier, France; 9 French National Platform Quality of Life and Cancer, Montpellier, France

**Keywords:** Health-related quality of life, Item response theory, Mixed models, Ordinal data, Longitudinal analysis

## Abstract

**Background:**

The use of health-related quality of life (HRQoL) as an endpoint in cancer clinical trials is growing rapidly. Hence, research into the statistical approaches used to analyze HRQoL data is of major importance, and could lead to a better understanding of the impact of treatments on the everyday life and care of patients. Amongst the models that are used for the longitudinal analysis of HRQoL, we focused on the mixed models from item response theory, to directly analyze raw data from questionnaires.

**Methods:**

We reviewed the different item response models for ordinal responses, using a recent classification of generalized linear models for categorical data. Based on methodological and practical arguments, we then proposed a conceptual selection of these models for the longitudinal analysis of HRQoL in cancer clinical trials.

**Results:**

To complete comparison studies already present in the literature, we performed a simulation study based on random part of the mixed models, so to compare the linear mixed model classically used to the selected item response models. As expected, the sensitivity of the item response models to detect random effects with lower variance is better than that of the linear mixed model. We then used a cumulative item response model to perform a longitudinal analysis of HRQoL data from a cancer clinical trial.

**Conclusions:**

Adjacent and cumulative item response models seem particularly suitable for HRQoL analysis. In the specific context of cancer clinical trials and the comparison between two groups of HRQoL data over time, the cumulative model seems to be the most suitable, given that it is able to generate a more complete set of results and gives an intuitive illustration of the data.

**Electronic supplementary material:**

The online version of this article (doi:10.1186/s12874-017-0410-9) contains supplementary material, which is available to authorized users.

## Background

In cancer clinical trials, endpoints refer to the biological and clinical measurements used to assess the efficiency of new therapeutic strategies. Overall survival is the gold standard endpoint used to show a clinical benefit of the strategies and treatments being trialed. However, therapeutic treatments are becoming more efficient, leading to an increase in patients’ lifespans, and therefore an overall survival endpoint may be insufficient to show a significant difference between two treatments. It is then necessary to consider a longer follow-up or a larger cohort of patients to have a sufficient number of events and a good statistical power [1], both representing considerable costs. Therefore, to assess the benefit of a new treatment, other endpoints have emerged, and health-related quality of life (HRQoL) is currently one of the most important, with HRQoL data routinely collected in cancer clinical trials. Patient-reported outcomes are being increasingly used in medical decision making to assess the clinical benefit of therapeutic treatments and strategies [1]. Moreover, the use of HRQoL as an endpoint may be more pertinent to demonstrate the benefit of a new therapy in some cases, such as for palliative or geriatric treatments.

In oncology, HRQoL is assessed using both a generic questionnaire and an additional specific questionnaire associated with each type of cancer [2, 3]. Each questionnaire breaks down the HRQoL to measure several underlying concepts (functional and symptomatic dimensions of HRQoL), which themselves comprise one or several items. The items are built on Likert scales, in which the response variable is ordinal. In European cancer clinical trials, the standard questionnaire used is the European organization for research and treatment of cancer Quality of Life Questionnaire - Core 30 (EORTC QLQ-C30) [2]. EORTC QLQ-C30 is composed of 30 ordinal items assessing several dimensions of HRQoL: the global health status (GHS), five functional dimensions (physical, role, cognitive, emotional and social), four multi-item symptomatic dimensions (fatigue, pain, nausea and vomiting, loss of appetite), and five single item symptomatic dimensions (diarrhea, constipation, insomnia, dyspnea and perceived financial impact). It is completed by the patients themselves, and collected at different time points defined in the trial protocol (usually at inclusion, during treatment and at follow-up). These repeated measurements are used to assess the evolution of the subjects’ HRQoL over time. According to the scoring procedure proposed by the EORTC [4], a score is then calculated for each dimension and for each subject at each time, corresponding to the average of the item responses for a single dimension, and expressed on a scale ranging from 0 to 100. The interpretation is such that high functional scores reflect good functional capacities and a good HRQoL level, and conversely, high symptomatic scores represent strong symptoms and point out difficulties. The use of scoring procedures is common practice because the statistical methods for quantitative variables are more powerful and easier to implement and interpret [5]. However, in a Likert scale, the gap which separates each adjacent category of response (“not at all”, “a little”, “quite a bit” and “very much”) may not be the same, and the calculation used to generate a HRQoL score does not take this characteristic into account. Another drawback to the HRQoL score is that subjects could have different item outcomes and obtain the same score. In this situation, the score does not make a distinction between these subjects [6].

The longitudinal statistical approach classically used in oncology is to apply a linear mixed model (LMM) to the patient score [7]. Mixed models take into account the correlation introduced by repeated measurements on the same patient (i.e. collection of the HRQoL questionnaires over time), and different covariates such as time, treatment group and age etc. However, the use of the LMM for HRQoL analysis is scientifically questionable. Since the variable associated with the HRQoL score is then considered as a continuous variable, whilst it presents the characteristics of an ordinal variable, being non-continuous and bounded. Furthermore, many symptomatic dimensions are composed of only one item, and the HRQoL score has exactly the same properties as ordinal categorical data, therefore using the LMM is not appropriated. Thus, if a ceiling or floor effect is observed, the categorical feature is even more marked when one of the two extreme categories is over-represented.

Interest in using HRQoL as an endpoint in cancer clinical trials is growing rapidly, hence it is essential to use a suitable methodology to analyze HRQoL data, taking into account the data properties (repeated measurements of multiple ordinal responses). In our work, we first focused on the different most adapted models used to analyze HRQoL from raw data, i.e. directly based on the item outcomes. Studies on psychometric properties from questionnaires such as the one used for HRQoL have been ongoing for a long time [8, 9], known as the item response theory (IRT). The IRT models link the individual’s item responses and a unique latent variable which represents the studied HRQoL concept. They can be seen as generalized linear mixed models (GLMM) for ordinal responses with a particular parameterization of the linear predictor. The interest in this kind of model to analyze data, including longitudinal analysis, is increasing [6, 10–12]. However, to our knowledge, there is no study that discusses the choice of one of the different IRT models over the others for the longitudinal analysis of HRQoL. First, we propose in the “Methods” section a conceptual selection of these models through practical and methodological arguments. For this, we replaced IRT models in the GLMM framework using the new specification of generalized linear models (GLM) for categorical responses, proposed by Peyhardi et al. [13]. Then, we carried out both a simulation study and an application on data from a cancer clinical trial in the “Results” section. As some previous simulations have compared IRT models and LMM on their capacity to detect fixed effects [7], we focused on the sensitivity of these models to detect random effects. The selected IRT model was then used to analyze real data from a multicenter randomized phase III clinical trial in first-line metastatic pancreatic cancer patients [14].

## Methods

This section concerns a conceptual selection of IRT models for the longitudinal analysis of HRQoL in cancer clinical trials. HRQoL raw data are repeated measurements of multiple ordinal responses. The GLMM for ordinal responses seem suitable to analyze this kind of data. The incorporation of random effects takes into account inter-patient variability and the correlation between repeated measurements for each single patient. IRT models turn out to be GLMM for polytomous data with a specific parameterization of the linear predictor, taking into account multiple outcomes. For ordinal responses, three families of regression models are described: adjacent models [15, 16], cumulative models [17, 18] and sequential models [19, 20]. Many IRT models are proposed for the analysis of this kind of data, often with no explanation regarding the choice of one model over another.

In this section, we used the new specification of the GLM for categorical data, as proposed by Peyhardi et al. [13], to discuss the relevance of the models adopted in the context of longitudinal analysis of HRQoL in cancer clinical trials. Whatever the model’s family, each GLM for categorical responses is defined according to three components *(r,F,Z)*: the ratio of probabilities (*r*), the cumulative distribution function (CdF) (denoted by *F*) and the parameterization of the linear predictor determined by the design matrix (*Z*). For the GLMM framework, we extended this new specification to the quadruplet *(r,F,Z,U)*, with *Z* being the design matrix of fixed effects, and *U* the design matrix of random effects. The relationship between these components is determined by $R=\mathcal {F}(Z\beta +U\xi)$. Given the linear predictor ***η***=*Z* *β*+*U* *ξ* and $\boldsymbol {\pi }_{iv}^{(j)}=\left (\pi _{iv0}^{(j)},\ldots,\pi _{ivM_{j}}^{(j)}\right)$ the vector of conditional probabilities with $\pi _{ivm}^{(j)}=\Pr \left (Y_{iv}^{(j)}=m\vert \xi _{i}\right)$ the conditional probability that subject *i*(*i*=1,…,*n*) selects the category $m\in \left \{0,\ldots,M_{j}\right \}$ for item *j*(*j*=1,…,*J*) at visit *v*(*v*=1,…,*n*_*i*_) given individual random effect, we defined:

$$R=\left\{r_{m}\left(\boldsymbol{\pi}_{iv}^{(j)}\right)\right\}_{i,j,v,m}, $$

and

$$\begin{aligned} \mathcal{F}\left\{\left({\eta}_{ivm}^{(j)}\right)_{i,j,v,m}\right\}&=\left\{F\left({\eta}_{ivm}^{(j)}\right)\right\}_{i,j,v,m}\\\text{where }\boldsymbol{\eta}&=\left({\eta}_{ivm}^{(j)}\right)_{i,j,v,m}. \end{aligned} $$

After a discussion of the IRT parameterization used concerning the linear predictor, we compare different polytomous IRT models on the basis of the link function (ratio of probabilities and the CdF), using both methodological and practical arguments.

### IRT parameterization of the linear predictor

The IRT probabilistic models emerged following the works of Georg Rasch [21] on dichotomous responses, and were then extended to ordinal responses. Considering the three families of adjacent, cumulative and sequential models, there are three associated famous IRT models [22, 23], respectively, the graded response model [17], the (generalized) partial credit model [15, 24] and the sequential model [19]. These models link the individual’s item responses to the unidimensional latent variable, which represents a concept not directly measurable. In an oncology setting, the concept is HRQoL relative to one specific HRQoL dimension.

In IRT, the specific parameterization of the linear predictor $\eta _{im}^{(j)}$ combines two parts: the individual part and the item part. This is most commonly defined using the following decomposition:

1 $$ \eta_{im}^{(j)}=\alpha_{j}\left(\theta_{i}-\delta_{jm}\right),  $$

where *θ*_*i*_ is associated with a unidimensional random variable (currently assumed to be distributed through the standard normal distribution for identifiability), representing the latent value for the *i*-th subject which quantifies the dependence between each item response, *δ*_*jm*_ and *α*_*j*_ being the item parameters which allow a fit of the model for each considered item. Generally denoted as the difficulty parameter, *δ*_*jm*_ is the threshold associated with the item *j* for the category $m\in \left \{1,\ldots,M_{j}\right \}$. The parameter *α*_*j*_ is known as the discrimination parameter of item *j*, and represents the sensitivity of each response probability according to the value of the latent trait. The higher the value of the discrimination parameter, the more the item allows for discriminating between two individuals with a close latent trait value. However, the predictor is no longer linear for IRT models using discrimination parameters, because it includes a product of parameters [25]. Therefore, these models do not belong to the class of GLMM.

In oncology, HRQoL analysis is classically carried out using IRT models which do not include discrimination parameters (fixed to one for all items). Consequently these IRT models are within the class of GLMM. Concerning longitudinal analysis, several studies have proposed to extend some IRT models using linear decomposition of the latent variable *θ* with fixed and random effects [26–28]:

2 $$ \theta_{iv}=x_{iv}'{\beta}+u_{v}'{\xi}_{i},   $$

where *β* is the parameter vector associated with fixed effects, *ξ*_*i*_ is the vector of the subject-specific random effects and *θ*_*iv*_ is thus the estimation of latent process at the visit *v*.

### The probability ratio: structure of the models

The ratio of probabilities is the component which defines membership to a particular family of models. Regarding categorical responses, the linear predictor is not directly related to the response probability, but to a particular transformation ratio. The choice of ratio is related to the nature of the response from the ordering assumption among categories. Thus, the reference ratio [13] for nominal responses is excluded in this work, because HRQoL responses are ordinal.

First, let us consider the simple situation from GLM with one item with (*M*+1) response categories given in the ascending order. The three model families for ordinal data are distinguished by the choice of the ratio of probabilities $\boldsymbol {r}\left (\boldsymbol {\pi }\right)=\left (r_{0}\left (\boldsymbol {\pi }\right),\ldots,r_{M-1}\left (\boldsymbol {\pi }\right)\right)$. Each model is summarized by *M* equations $\left \{r_{m}\left (\boldsymbol {\pi }\right)=F\left (\eta _{m}^{\star }\right)\right \}_{m=0,\ldots,M-1}$, highlighting the decomposition of the link function, which is determined through the ratio of probabilities and the CdF. Indeed, we may distinguish different ratios of probabilities for these different families, respectively, for the cumulative models,

3 $$\begin{array}{@{}rcl@{}} r_{m}\left(\boldsymbol{\pi}\right) = \pi_{0}+\ldots +\pi_{m},\quad m=0,\ldots,M-1;  \end{array} $$

for the adjacent models,

4 $$\begin{array}{@{}rcl@{}} r_{m}\left(\boldsymbol{\pi}\right)=\frac{\pi_{m}}{\pi_{m}+\pi_{m+1}},\quad m=0,\ldots,M-1;  \end{array} $$

and, for the sequential models,

$$\begin{array}{@{}rcl@{}} r_{m}\left(\boldsymbol{\pi}\right)=\frac{\pi_{m}}{\pi_{m}+\ldots +\pi_{M}},\quad m=0,\ldots,M-1. \end{array} $$

In IRT, adjacent and cumulative models are usually presented given the reverse permutation [15, 17, 23]. This permutation is defined as the reversal of category order [18]. Assuming that the considered CdF is symmetric (i.e. the coresponding probability density function is symmetric about the y-axis), these models are invariant under this permutation [13]. In the context of our application, this is an advantage for the interpretation of the results. A lower item-response category reflects a lower level of capacity for the symptomatic dimensions, whereas it represents a higher level of capacity for the functional dimensions. A reverse permutation of the functional dimensions, makes it easier and more intuitive for clinicians to present their results. This allows for homogenization in the interpretation of results, as is present in the scoring procedure proposed by the EORTC (for functional dimensions the score scale is reversed compared with the order of the item response categories) [4]. Since HRQoL data is from a ordered scale, both the adjacent and cumulative models are suitable. However, sequential models are not reversible, because they correspond to process ordering, and reversing the process may change its nature. Thus, sequential models will not be used, and only the adjacent and cumulative models, which correspond to scaled ordering (as used for HRQoL measurements), will be considered.

From now on, we consider the simple situation from GLM, with one item with (*M*+1) response categories given in the descending order as commonly seen in IRT. Then, $\boldsymbol {r}\left (\boldsymbol {\pi }\right)=\left (r_{1}\left (\boldsymbol {\pi }\right),\ldots,r_{M}\left (\boldsymbol {\pi }\right)\right)$, where the model is summarized by *M* equations $\left \{r_{m}\left (\boldsymbol {\pi }\right)=F\left (\eta _{m}\right)\right \}_{m=1,\ldots,M}$ with *η*_*m*_=*θ*−*δ*_*m*_. The ratio of probabilities defined in Eqs. (3) and (4) are given in descending order by:

5 $$\begin{array}{@{}rcl@{}} r_{m}\left(\boldsymbol{\pi}\right) = \pi_{m}+\ldots +\pi_{M},\quad m=1,\ldots,M;  \end{array} $$

for the cumulative models and by:

$$\begin{array}{@{}rcl@{}} r_{m}\left(\boldsymbol{\pi}\right)=\frac{\pi_{m}}{\pi_{m}+\pi_{m-1}},\quad m=1,\ldots,M; \end{array} $$

for the adjacent models. Peyhardi et al. [13] described the transformation between the linear predictors $\eta _{m}^{\star }$ and *η*_*m*_, for ascending and descending orders, respectively. Therefore, the probabilities for the cumulative model are defined from the Eq. (5) and given *F* as:

6 $$ \left\{ \begin{array}{ccl} \pi_{0} & = & 1- F\left(\eta_{1}\right)\\ \pi_{m} & = & F\left(\eta_{m}\right) - F\left(\eta_{m+1}\right),\quad m=1,\ldots,M-1\\ \pi_{_{M}} & = & F\left(\eta_{_{M}}\right) \end{array} \right..  $$

In the literature, the cumulative model is associated with the use of several of the previously mentioned CdF [17, 20, 25], whilst the adjacent models are only associated with logistic CdF [7, 15, 16, 20, 24, 26]. However, the different response probabilities can be presented from the adjacent ratio and according to a general CdF (*F*):

7 $$  \left\{ \begin{aligned} \pi_{0} & = & \frac{1}{1+\sum_{m=1}^{M}\prod_{k=1}^{m}\left(\frac{F(\eta_{k})}{1-F(\eta_{k})}\right)}\\ \pi_{m} & = & \frac{\prod_{k=1}^{m}\left(\frac{F(\eta_{k})}{1-F(\eta_{k})}\right)}{1+\sum_{m=1}^{M}\prod_{k=1}^{m} \left(\frac{F(\eta_{k})}{1-F(\eta_{k})}\right)}&,\quad m=1,\ldots,M \end{aligned} \right.  $$

The cumulative models also have additional properties [18], including that they are invariant when successive categories are gathered. Thus, if one category is not observed, it can be combined with its successive categories without changing the model. Another advantage of the cumulative models is their interpretation through a continuous latent response variable $\widetilde {Y}$. Indeed, this latent variable underlying the model exists and a direct link with the response variable *Y* through the thresholds presumed to be strictly increasing ($-\infty =\delta _{0}<\delta _{1}<\ldots <\delta _{M}<\delta _{M+1}=+\infty $) is such as:

$$\begin{array}{@{}rcl@{}} \left\{Y=m\right\} & \text{if} & \left\{\delta_{m}<\widetilde{Y}\leq\delta_{m+1}\right\},\quad m=0,\ldots,M\;, \end{array} $$

where $\widetilde {Y}=\theta +\varepsilon $ and *ε* is the error term distributed following the CdF. Here, the latent variable $\tilde {Y}$ represents HRQoL and its interpretation is then equivalent to the one of the response variable using a LMM.

An advantage of the adjacent models is that there are no constraints affecting the model estimation. However, the cumulative models have to respect constraints, which can make model estimation difficult, particularly in the case of a non-proportional design of the linear predictor [13]. For the proportional design, a common variable *θ* is considered for all categories, otherwise it is dependent on the category (*θ*_*m*_). Considering a proportional design (*θ*=*θ*_1_=…=*θ*_*M*_), the cumulative models refer to the principle of thresholds [18, 29], with the constraint that they have to be strictly increasing such as $-\infty <\delta _{1}<\ldots <\delta _{M}<+\infty $. Considering the non-odd proportional models, the constraint then becomes $-\infty <\eta _{M}<\ldots <\eta _{1}<+\infty $, which is more difficult to verify.

Table 1 summarizes some of the characteristics of the three families of models which are considered important for the longitudinal analysis of HRQoL in cancer clinical trials. In this context, a proportional design of the linear predictor is classically used. Under this parameterization, the cumulative model’s constraints are only on the threshold, making easier to estimate these models. Moreover, the cumulative models’ interpretation utilizing the underlying continuous latent response variable, which directly links the observed outcomes through threshold parameters, given a more intuitive interpretation of results than is achieved using the adjacent models. Despite the fact the cumulative model is more appropriate, the adjacent model is more flexible because there are no constraints to verify. Therefore, in another context with non-proportional design, the adjacent model may be preferred.

**Table 1:** Summary of the characteristics for the three model families

	Models		
Characteristics	Adjacent	Cumulative	Sequential
Ordinal scale	*yes*	*yes*	*no*
Reversibility	*yes*	*yes*	*no*
Interpretation using the latent variable	*no*	*yes*	*yes*
Always defined	*yes*	*yes*(*no* ^a^)	*yes*

^a^for some non proportional design models

### The cumulative distribution function

The last component of the IRT model selection to be discussed is the CdF. Each model probability can be defined with any CdF and the choice of which CdF to use should be that which best fits the data. Let’s consider four CdF from two different kinds: the most commonly used symmetric distributions, the logistic and Gaussian distributions, and the two asymmetric distributions, the Gumbel min and Gumbel max distributions. The two later distributions are respectively defined by *F*(*η*)=*exp*(−*exp*(−*η*)) for the Gumbel max distribution and by *F*(*η*)=1−*exp*(−*exp*(*η*)) for the Gumbel min distribution.

**Fig. 1:**
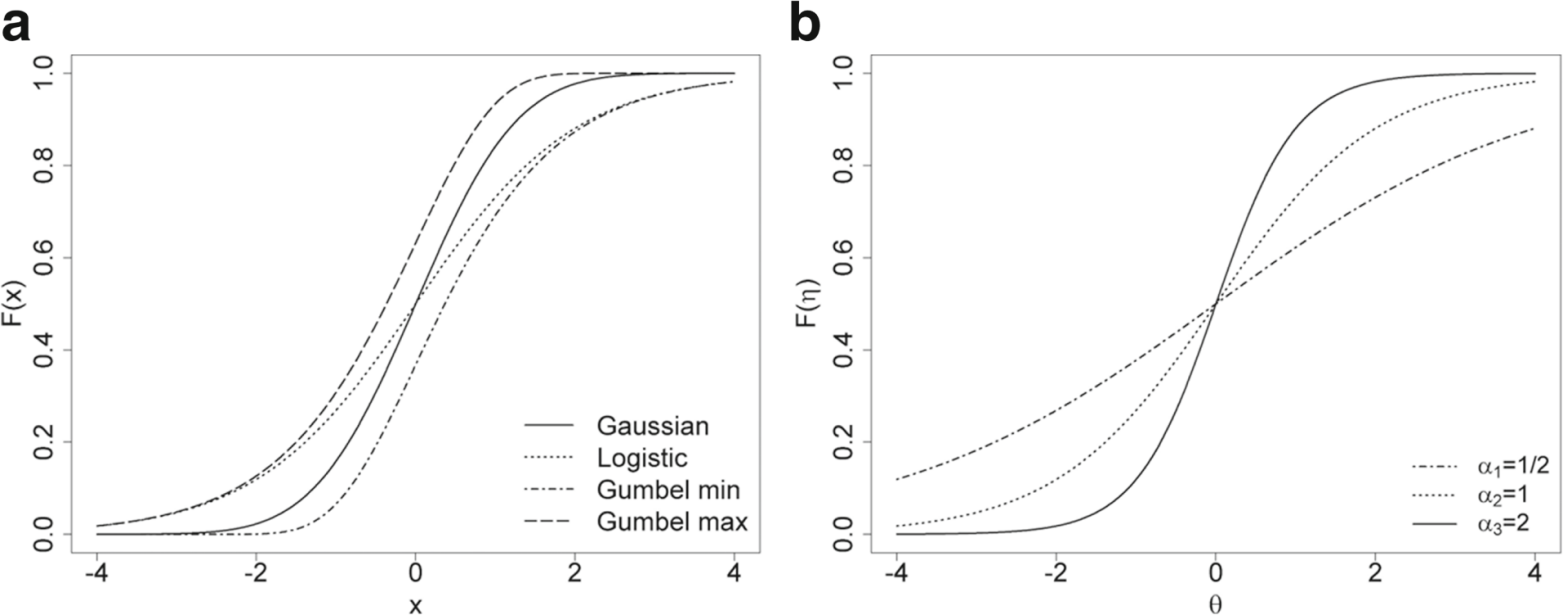
Relationship between the CdF and the IRT parameterization for dichotomous items where *F*(*η*)=*F*(*α*_*j*_(*θ*−*δ*_*j*_)). **a** presents the different CdF for one item *j* given *α*_*j*_=1 and *δ*_*j*_=0. **b** presents the logistic CdF adjustment for three items (*j*=1,2,3) with different *α*_*j*_ and *δ*_*j*_=0, given the linear predictor defined in Eq. (1)

Figure 1 a shows different slopes depending on the particular CdF. The CdF allows to take into account the influence of linear predictor (*η*) change on the response probability evolution. In general IRT parameterization (Eq. 1), the slope adjustment is managed by the discrimination parameter. Depending on different discrimination parameter values, Fig. 1 b shows the CdF logistic according to the individual latent variable. This item parameter has the task of fitting the CdF slope for each considered item. In the context of HRQoL in clinical trials, the HRQoL dimension considers a small set of items which are correlated, and measures a unique latent variable. The discrimination parameter is not routinely used in this kind of analysis. Moreover, the use of a symmetric CdF seems more suitable given the tendency to use reversible models in the context of the HRQoL in clinical trial.

Relative to the literature, Table 2 outlines the specifications and the different components of the famous polytomous IRT models. For IRT models within the class of GLMM, we propose to define them using the four components *(r,F, Z*_*q*_,*U*_*a*_ *)*. The kind of considered location item parameters can be indicated by the index *q*, where *q*=1 when including only difficulty parameters. Let *q*=2 when considering the rating scale model [30] parameterization, where difficulty parameters are common for all items and one shift parameter is considered for each item. Regarding the random part, the number of random effects is indicated by the index *a*. For the classical IRT parameterization presented in Table 2, only one random effect (*r*=1) is taken into account: the latent variable *θ*. For IRT models including discrimination parameters for each item, we proposed to replace the components *Z* and *U* by a component specifying that the predictor is no longer linear (nl), such as *(r,F,nl)*.

**Table 2:** Specification of the famous IRT model following the components: *(r,F, Z*_*q*_,*U*_*a*_ *)* for the GLMM and *(r,F,nl)* for IRT model with no longer linear predictor

IRT models	$\eta _{im}^{(j)}$	*(r,F, Z*_*q*_,*U*_*a*_ *)*
Rating scale model	$\theta _{i}-\left (\delta _{m}+\tau _{j}\right)$	*(adjacent,logistic,* *Z*_2_,*U*_1_ *)*
Partial credit model	*θ*_*i*_−*δ*_*jm*_	*(adjacent,logistic,* *Z*_1_,*U*_1_ *)*
Sequential Rasch model	*θ*_*i*_−*δ*_*jm*_	*(sequential,logistic,* *Z*_1_,*U*_1_ *)*
Graded response model	$\alpha _{j}\left (\theta _{i}-\delta _{jm}\right)$	*(cumulative,logistic,nl)*
Generalized partial credit model	$\alpha _{j}\left (\theta _{i}-\delta _{jm}\right)$	*(adjacent,logistic,nl)*

Index *q* denotes the number of kind of item parameters considered in the IRT model and *a* the number of random effects

### Software

Simulation and application studies were performed using the SAS procedure *PROC NLMIXED* from the SAS software (version 9.3) [22, 31]. SAS codes to estimate IRT adjacent and cumulative models are available in the Additional file 1.

## Results

### Simulation study

In the previous section, we focused on the use of mixed models for ordinal data analysis, and discussed their relevance in the HRQoL analysis in oncology. Some previous studies have compared IRT models to the classical approaches (in particular the LMM) [7, 32, 33], mainly focusing on the fixed part of the mixed models to identify trends in latent traits. For example, Anota et al. [7] show an equivalent capacity of both the LMM and one of the IRT models to detect a fixed effect. Indeed, even if the LMM take into account the HRQoL score, which is a summary variable, this approach is at least equivalent to the IRT models in terms of power.

In our simulation study, the adjacent and cumulative models used the same parameterization of the linear predictor and the logistic CdF (as is usual for longitudinal analysis with IRT models). The aim of the following section is to reinforce comparisons between the LMM and the IRT models on the random part of the mixed models. The datasets were simulated from an IRT model (adjacent and cumulative models). Regarding the parameterization, two subject-specific random effects *ξ*_*i*0_ and *ξ*_*i*1_ were considered, respectively associated with the intercept and the slope. Of course, the usefulness of introducing random effects to the model is strongly dependent upon the observed data. HRQoL is a subjective endpoint, and the inclusion of individual random effect *ξ*_*i*0_ is thus entirely justified. Indeed, it is easy to imagine that each patient has a different level of HRQoL at baseline. The inclusion of the random slope is more questionable, indeed, the assumption that the specific HRQoL evolution of one single patient diverges from the average evolution for the whole population is less obvious than the previous assumption that each patient has a different level of HRQoL at baseline. Thus, in this section, we studied the capacities of the adjacent and cumulative mixed models to detect the random slope.

#### Design

We aimed to study the capacity of each model to detect the random effect *ξ*_*i*1_ associated with time (random slope). The two subject-specific random effects are considered independent where $\xi _{i0}\sim \mathcal {N}\left (0,\sigma _{0}^{2}\right)$ and $\xi _{i1}\sim \mathcal {N}\left (0,\sigma _{1}^{2}\right)$. The following model choice study is performed on the basis of the Bayesian information criteria (BIC) where two models were considered: $\mathcal {M}_{2}$ with the two random effects *(r,F,* *Z*_1_,*U*_2_ *)* and $\mathcal {M}_{1}$ excluding the random slope *(r,F,* *Z*_1_,*U*_1_ *)*. For the IRT models, the linear decomposition of the latent trait *θ*_*iv*_ only took into account time as a fixed effect. The two considered models with proportional design are:

8 $$ \left. \begin{aligned} \mathcal{M}_{2}: \theta_{iv} & = \left(t_{v}-t_{0}\right)\beta_{1}+\xi_{i0}+\left(t_{v}-t_{0}\right)\xi_{i1}\\ \mathcal{M}_{1}: \theta_{iv} & = \left(t_{v}-t_{0}\right)\beta_{1}+\xi_{i0} \end{aligned} \right.   $$

In order to best reflect the EORTC QLQ-C30 questionnaire, the most frequent HRQoL dimension with two items (*j*=1,2) comprising four response categories ($m\in \left \{0,\ldots,M\right \}$ with *M*=3), was used to design the simulation study. A sample size of 300 subjects (*i*=1,…,*n* with *n*=300) with eight follow-up time points *t*=(0,0.5,1,2,4,6,8,10) was used. The datasets were simulated from a multinomial distribution. The different response probabilities $\left \{\pi _{ivm}^{(j)}=\Pr \left (Y_{iv}^{(j)}=m\vert \theta _{iv},\delta _{j}\right)\right \}$ concerning the subject *i* for item *j* were determined by Eq. (7) for the adjacent model and by Eq. (6) for the cumulative model, given: the item parameters $\delta _{j}=\left (\delta _{j1},\delta _{j2},\delta _{j3}\right)_{j=1,2}$, the latent trait (*θ*_*iv*_) deduced in accordance with Eq. (8), and the logistic CdF,

$$F\left(\eta_{ivm}^{(j)}\right)=\frac{\exp\left(\eta_{ivm}^{(j)}\right)}{1+\exp\left(\eta_{ivm}^{(j)}\right)}, $$

where $\eta _{ivm}^{(j)}=\theta _{iv}-\delta _{jm}$.

The values of the parameters used were deduced from the pain symptom data from the clinical trial presented in the application subsection. We considered two kinds of difficulty parameters: near ${\delta ^{ne}=\left (\delta _{1}^{ne},\delta _{2}^{ne}\right)}$ and far ${\delta ^{fa}=\left (\delta _{1}^{fa},\delta _{2}^{fa}\right)}$. These parameter values were chosen in order to illustrate the several scenarios described in Table 3. The different scenarios were due to the different associations between the model used to simulate the data, *(adjacent,logistic,* *Z*_1_,*U*_*a*_ *)*_*a*=1,2_ or *(cumulative,logistic,* *Z*_1_,*U*_*a*_ *)*_*a*=1,2_, and the different considered values of the difficulty parameters. Table 3 shows the simulated responses expected at baseline (*t*=0). The responses simulated across time depend on of the considered coefficient, *β*_1_. Each scenario was simulated *N*=500 times.

**Table 3:** Values of difficulty parameters used to simulate the data and expected responses at *t*_0_ under each studied scenarios

	Difficulty parameters	
Models	$\delta _{1}^{ne}=(-1.6,1,1.45)$	$\delta _{1}^{fa}=(-2.1,1,2.75)$
(*r,F,Z*_1_,*U*_*a*_)_*a*=1,2_	$\delta _{2}^{ne}=(-0.8,1.15,1.9)$	$\delta _{2}^{fa}=(-1.25,1.4,3.3)$
*(adjacent,logistic,* *Z*_1_,*U*_*a*_ *)*_*a*=1,2_	Balanced responses	Focus on center categories (1 and 2)
*(cumulative,logistic,* *Z*_1_,*U*_*a*_ *)*_*a*=1,2_	Focus on extreme categories (0,1 and 3)	Balanced responses

Concerning the LMM, the scoring procedure proposed by the EORTC was considered [4], and the score associated with a symptomatic dimension was first calculated using the simulated data. Considering the two simulated ordinal outcomes $y_{iv}^{(1)}$ and $y_{iv}^{(2)}$ concerning the individual *i* at the visit *v*, the related score was:

$$S_{iv}=\left(\frac{\sum_{j=1}^{J=2}y_{iv}^{(j)}}{2}\right) \frac{100}{M}. $$

Similar to the parameterization in Eq. (8), we took into account the related choice model with:

9 $$ \begin{aligned} \mathcal{M}_{2}: S_{iv} & = \beta_{0}^{^{l}} + \left(t_{v}-t_{0}\right)\beta^{^{l}}_{1}+\xi^{^{l}}_{i0}+\left(t_{v}-t_{0}\right)\xi^{^{l}}_{i1} + \varepsilon_{iv}\\ \mathcal{M}_{1}: S_{iv} & = \beta_{0}^{^{l}} + \left(t_{v}-t_{0}\right)\beta_{1}^{^{l}}+\xi^{^{l}}_{i0} +\varepsilon_{iv} \end{aligned}  $$

where $\beta _{0}^{^{l}}$ is the fixed parameter associated with the intercept, $\xi ^{^{l}}$ are the random effects normally distributed with the mean equal to zero, and $\varepsilon _{iv}\sim \mathcal {N}\left (0,\sigma ^{2}_{\varepsilon }\right)$ the error term.

#### Simulation results

Table 4 shows the capacity of the three models (adjacent model, cumulative model and LMM) to detect the random slope in different given scenarios (Table 3). When we simulated the data under $\mathcal {M}_{2}$ according to the random effect variances estimated from real data, each model detected the random slope (*ξ*_*i*1_) in 100% of cases whatever the given situation. As expected under $\mathcal {M}_{1}$, the simulated model $\mathcal {M}_{1}$ was correctly chosen in most cases, and in particular for the IRT model used to generate the datasets. However, for all simulations under $\mathcal {M}_{1}$, the cumulative model seemed to detect the random slope in about 5 to 10% of cases, although it was not included in the simulation step. Moreover, the IRT model which was not used to generate the data, wrongly detected this random effect given a negative value of *β*_1_ and the difficulty parameter coefficients *δ* ^*ne*^. This is caused by the relationship between the latent variable *θ* which changes over time and *δ* ^*ne*^ which accounts for the observed ordinal responses over time. For these specific parameter values (*β*_1_≈−0.3 and *δ* ^*ne*^ given in Table 3), the linear predictors $\eta _{itm}^{(j)}=\theta _{it}-\delta ^{ne}_{jm}$ were close between them for *m*=2,3 whatever *j*=1,2. These linear predictors being negative and different from zero value, the probability of selecting the upper categories was very small over time and under-represented in comparison to the lower categories. In this specific case, the IRT model used to generate the data had the advantage of being closest to the data and only required the use of the fixed effect and the random intercept to explain the different outcomes, whereas the other model compensated by using the random slope. We then could expect symmetric results from *β*_1_ (positive values), considering the opposite sign of the difficulty parameters, because of the reversibility property of the IRT models given symmetric CdF. On the contrary, the LMM was stable and thus proved to be a choice of model whatever the *β*_1_ values and the IRT model used to simulate the data.

**Table 4:** Percentages of selecting $\mathcal {M}_{1}$ according to the BIC on *N*=500 datasets, given *t*_*v*_=(0,1,2,4,6,8,10,12) and $\sigma _{0}^{2}=1.5$

Parameter		Scenarios											
Values		AM using *δ* ^*ne*^			CM using *δ* ^*fa*^			CM using *δ* ^*ne*^			AM using *δ* ^*fa*^		
$\sigma _{1}^{2}$	*β*_1_	LMM	AM	CM	LMM	AM	CM	LMM	AM	CM	LMM	AM	CM
0.2	−0.3	0	0	0	0	0	0	0	0	0	0	0	0
0.2	0.3	0	0	0	0	0	0	0	0	0	0	0	0
0	−0.5	97.7	99.3	56.49	100	94.6	93.0	100	61.3	95.7	100	99.7	89.5
0	−0.3	99.0	100	33.0	100	88.6	93.3	100	36.3	94.9	100	100	83.3
0	−0.2	100	99.6	49.3	100	94.6	93.8	100	71.7	95.8	100	99.6	79.0
0	−0.1	98.7	95.7	94.8	100	98.7	89.6	100	99.0	90.4	100	100	88.1
0	0.0	95.6	100	94.6	99.0	99.7	91.8	99.0	99.7	89.7	97.0	99.7	94.4
0	0.1	83.0	100	94.8	93.3	100	92.6	97.0	100	90.9	87.3	100	94.7
0	0.3	98.3	99.6	90.6	100	99.6	89.1	100	100	93.7	100	99.6	93.8
0	0.5	100	100	94.3	100	99.3	94.7	100	100	97.6	100	100	97.2

The *(adjacent,logistic,* *Z*_1_,*U*_*a*_ *)*_*a*=1,2_ models and the *(cumulative,logistic,* *Z*_1_,*U*_*a*_ *)*_*a*=1,2_ models are denoted respectively by AM and CM. For the random component, *U*_1_ if $\sigma _{1}^{2}=0$ and *U*_2_ if $\sigma _{1}^{2}>0$

The capacity of the different models to detect the random slope when its variance value changes is presented in Table 5. Only the values of $\sigma _{1}^{2}$ for which the capacities varied between the three models are presented for each considered value of *β*_1_. Each model was sensitive to the signal-to-noise ratio: the larger the value of |*β*_1_|, the larger the variance of the random effect needed to be detected. For example, when |*β*_1_|=1, each model detected the random slope at 100 percent for $\sigma _{1}^{2}$ being over 0.5, while they detected it for $\sigma _{1}^{2}$ being over 0.2 when |*β*_1_|=0.3. When the models were compared, the IRT models showed a better capacity than the LMM to detect the random effect with small variance, whatever the value of *β*_1_. Moreover, the capacity of the IRT models remained stable for the different given scenarios, whilst the LMM’s changed. For *β*_1_=0.3 and *β*_1_=0 (cases where a lot of different higher responses were observed), the capacity of the LMM was close to that of the IRT models, whilst for the other scenarios, the capacity of the LMM was lower. Comparing the two IRT models, there is a tendency for the random slope model to be preferred under the cumulative model regardless of whether it is the true model or not. On the contrary, in the specific case where *β*_1_=−0.3, the IRT model used to simulate the data was less efficient than the other IRT model which detected a random slope to remedy the lack of information. This was consistent with our previous results shown in Table 4.

**Table 5:** Percentages of selecting $\mathcal {M}_{2}$ according to the BIC on *N*=500 datasets, given *t*_*v*_=(0,1,2,4,6,8,10,12) and $\sigma _{0}^{2}=1.5$

Parameter		Scenarios											
Values		AM using *δ* ^*ne*^			CM using *δ* ^*fa*^			CM using *δ* ^*ne*^			AM using *δ* ^*fa*^		
*β*_1_	$\sigma _{1}^{2}$	LMM	AM	CM	LMM	AM	CM	LMM	AM	CM	LMM	AM	CM
1	0.01	0	2.3	24.9	0	5.0	6.9	0	2.7	3.7	0.3	6.4	24.8
	0.02	0	21.4	54.7	0	37.6	44.1	0	17.7	18.1	0	50.0	77.0
	0.03	0	61.0	91.0	0	75.7	80.0	0	41.3	45.6	0	86.3	98.3
	0.05	0	97.7	99.7	0	100	100	0.3	89.0	90.0	0	99.3	100
	0.2	39.3	100	100	40.7	100	100	10.7	100	100	57.7	100	100
	0.5	100	100	100	100	100	100	100	100	100	100	100	100
0.5	0.005	0.2	25.5	56.4	0	41.2	25.7	0	14.9	11.0	0	41.2	53.6
	0.008	0.8	73.8	89.4	0	85.8	73.4	0	42.9	38.8	0.2	91.6	93.6
	0.01	2.0	91.2	97.0	0	97.0	91.6	0	66.6	63.4	0.6	99.2	99.2
	0.02	26.4	100	100	4.8	100	100	0	100	100	51.8	100	100
	0.03	77.0	100	100	64.8	100	100	0.8	100	100	96.6	100	100
	0.05	99.8	100	100	100	100	100	62.3	100	100	100	100	100
0.3	0.002	16.7	6.3	21.4	0	2.1	4.0	0	3.1	3.9	11.0	11.0	15.3
	0.005	72.3	86.3	92.7	30.7	55.3	59.0	0	32.3	46.0	85.7	87.3	91.7
	0.008	97.7	100	100	86.0	97.3	98.0	4.0	76.3	88.3	99.3	99.7	100
	0.01	100	100	100	96.3	99.7	99.3	17.3	94.0	97.0	100	100	100
	0.02	100	100	100	100	100	100	96.7	100	100	100	100	100
0	0.001	24.8	2.8	5.7	6.8	0.4	1.6	4.8	0.6	1.9	15.2	1.4	3.7
	0.002	70.2	32.0	37.3	26.4	6.6	8.2	20.6	2.4	5.1	47.6	15.2	21.4
	0.005	99.8	99.4	99.6	92.2	70.4	77.2	88.2	61.8	72.4	99.6	97.8	97.8
	0.008	100	100	100	99.8	98.0	98.8	99.8	98.4	99.2	100	100	100
	0.01	100	100	100	100	100	100	100	100	100	100	100	100
	0.02	100	100	100	100	100	100	100	100	100	100	100	100
−0.3	0.002	0.7	4.4	61.4	0	54.0	5.1	0	93.3	1.8	0	2.1	18.6
	0.005	5.7	62.3	79.0	0	95.7	40.4	0	99.7	33.2	0	56.0	48.3
	0.008	23.7	96.3	97.3	0	100	86.7	0	100	82.7	1.7	96.3	86.3
	0.01	45.2	100	99.6	0	100	98.2	0	100	92.3	6.8	99.8	98.4
	0.02	98.8	100	100	61.4	100	100	26.6	100	100	96.0	100	100
−0.5	0.005	2.6	12.1	48.6	0	57.6	13.2	0	84.8	9.8	0	41.2	53.6
	0.008	3.8	43.5	70.7	0	85.8	41.6	0	96.1	33.5	0.2	91.6	93.6
	0.01	5.6	70.0	84.8	0	95.6	61.5	0	98.4	49.6	0.6	99.2	99.2
	0.02	12.8	100	100	0	100	100	0	100	97.8	51.8	100	100
	0.03	36.8	100	100	0	100	100	0	100	100	96.6	100	100
	0.05	93.0	100	100	43.4	100	100	17.6	100	100	100	100	100
−1	0.01	0	0.6	34.5	0	5.8	5.2	0	8.4	6.3	0	1.6	15.1
	0.02	0	5.8	46.6	0	21.2	18.4	0	20.8	11.0	0	8.8	34.3
	0.03	0	30.4	73.2	0	50.4	44.8	0	50.0	37.4	0	36.4	58.0
	0.05	0	83.6	95.2	0	92.6	91.4	0	85.6	80.1	0	90.0	96.0
	0.2	46.4	100	100	21.4	100	100	12.0	100	100	41.8	100	100
	0.5	100	100	100	100	100	100	100	100	100	100	100	100

For the *(adjacent,logistic,* *Z*_1_,*U*_*a*_ *)*_*a*=1,2_ models and the *(cumulative,logistic,* *Z*_1_,*U*_*a*_ *)*_*a*=1,2_ models are denoted by AM and CM, respectively. For the random component, *U*_1_ if $\sigma _{1}^{2}=0$ and *U*_2_ if $\sigma _{1}^{2}>0$

In conclusion, the closer the value of *β*_1_ to zero (small signal), the easier it is for the models to detect the random slope with a low variance. The IRT models are more sensitive and stable than the LMM whatever the parameter values. This result was expected because the LMM is based on the HRQoL score, which is a summary variable with less information than the raw data. Comparing the IRT models, the one which was not used to generate the data tended to wrongly detect a random effect where there was none.

### Application to a real dataset

The real dataset we used was HRQoL data from a multicenter randomized phase III clinical trial in first-line metastatic pancreatic cancer patients: PRODIGE4/ACCORD11 [14]. Three hundred and forty-two patients were randomly assigned to Folfirinox (experimental arm) versus Gemcitabine (control arm) regimens. Detailed inclusion and exclusion criteria, study design and protocol, treatment, compliance to the questionnaires and HRQoL analysis have all previously been described [14, 33, 34]. The patients completed the EORTC QLQ-C30 questionnaire themselves at different follow-up times as defined in the protocol: at baseline, day 15, day 30, and at months 2, 4, 6, 8, and 10. The different time points reflect the longitudinal aspect of HRQoL and allow us to assess the change in HRQoL for each dimension.

Previously, cumulative models have been preferred for the longitudinal analysis of HRQoL, then the *(cumulative,logistic,* *Z*_1_,*U*_2_ *)* model is used to analyze data in this application. In oncology, analysis is carried out for each HRQoL dimension. Given one HRQoL dimension with few correlated items, the discrimination parameters could be considered equal to one for each item. Distinction between multiple-item responses is only achieved through the use off difficulty parameters (thresholds) [7, 33]. Given the subject $i\left (i=1,\ldots,342\right)$, the visit $v\left (v=1,\ldots,8\right)$, the item *j* with *M*_*j*_ response categories, the *(cumulative,logistic,* *Z*_1_,*U*_2_ *)* model is defined by:

10 $$ \Pr\left(Y^{(j)}_{iv}\geq m\vert \theta_{i}\right) = \frac{exp\left(\eta^{(j)}_{ivm} \right)}{1+exp\left(\eta^{(j)}_{ivm} \right)},  $$

with the following linear predictor considered in the analysis:

11 $$ \left\{ \begin{aligned} \eta_{ivm}^{(j)} =\;\; & \theta_{iv}-\delta_{jm} \\ \theta_{iv} =\;\; & g_{i}\beta_{1} + \left(t_{v}-t_{0}\right)\beta_{2}+g_{i}\left(t_{v}-t_{0}\right)\beta_{3}\\ & +\xi_{i0}+\left(t_{v}-t_{0}\right)\xi_{i1} \end{aligned} \right.   $$

where *δ*_*jm*_ is the difficulty parameter (threshold) associated with the category *m* of item *j*, *t*_*v*_ is the date of the visit *v*, and *t*_0_ is the date of baseline, *g*_*i*_=1 if the patient *i* belongs the experimental group (Folfirinox), *g*_*i*_=0 if the patient *i* belongs the control group (Gemcitabine), *β*_1_ is the effect difference at baseline between Folfirinox and Gemcitabine groups, *β*_2_ is the slope (evolution) of HRQoL perception for the Gemcitabine group, *β*_2_+*β*_3_ is the slope (evolution) of HRQoL perception for the Folfirinox group, and *ξ*_*i*0_ and *ξ*_*i*1_ are respectively the subject-specific random effects associated with the intercept and the slope such as $\left (\xi _{i0},\xi _{i1}\right)'\sim \mathcal {N}\left (\mathbf{0},\Sigma \right)$, *Σ* being the unstructured covariance matrix.

These HRQoL data have previsouly been analyzed using different approaches. Specifically, Gourgou-Bourgade et al. [34] analyzed the results using time-to-event models. They concluded HRQoL was better in the Folfirinox arm than in the Gemcitabine arm. Then, Barbieri et al. [33] have presented the results through the LMM and the partial credit model extended for the longitudinal analysis *(adjacent,logistic,* *Z*_1_,*U*_2_ *)*. The conclusions of both mixed models are similar.

For the *(cumulative,logistic,* *Z*_1_,*U*_2_ *)* model, Table 6 shows the estimations of fixed parameters, their standard deviation and the associated P-value from the Wald test. Concerning the functional dimension, we performed a reverse permutation on the functional scale for an intuitive interpretation. This allows us to consider that an increase in the latent variable *θ* is associated with an increase in the functional capacity (improvement of HRQoL) or an increase in the symptoms (deterioration of HRQoL). For all HRQoL dimensions, there should be no difference at baseline (*β*_1_=0) in a randomized clinical trial. However, we observed a significant difference in terms of diarrhea symptoms between the two groups at baseline (*P-* *value*=0.007^∗∗^). This is caused by an observed difference between the two arms of the study during the treatment period (at day 15 and day 30). This result was expected because Folfirinox is known as being more toxic than Gemcitabine, and also known to cause more diarrhea symptoms. Given our model does not take into account a possible difference between the two treatments during only this period, the fixed intercept was affected. The perception of diarrhea symptoms remained higher in the Folfirinox arm over time, particularly during the treatment period.

**Table 6:** Estimations of fixed effect parameters (*β*_*p*_)_*p*=1,2,3_ of the *(cumulative,logistic,* *Z*_1_,*U*_2_ *)* model

HRQoL				
Dimensions		Coefficient	Standard error	*P-value*
Global Health Status				
	*β*_2_	0.098	0.070	0.166
	*β*_3_	0.130	0.085	0.128
Physical functioning				
	*β*_2_	-0.150	0.077	0.051
	*β*_3_	0.122	0.098	0.212
Role functioning				
	*β*_2_	-0.011	0.081	0.892
	*β*_3_	0.157	0.103	0.131
Emotional functioning				
	*β*_2_	0.335	0.070	<.001^∗∗∗^
	*β*_3_	0.001	0.086	0.992
Cognitive functioning				
	*β*_2_	-0.002	0.054	0.972
	*β*_3_	0.088	0.067	0.189
Social functioning				
	*β*_2_	0.010	0.073	0.888
	*β*_3_	0.116	0.093	0.211
Fatigue				
	*β*_2_	-0.087	0.085	0.308
	*β*_3_	-0.033	0.107	0.761
Nausea and vomiting				
	*β*_2_	-0.052	0.060	0.393
	*β*_3_	-0.069	0.072	0.336
Pain				
	*β*_2_	-0.330	0.076	<.001^∗∗∗^
	*β*_3_	-0.188	0.092	0.040 ^∗^
Dyspnea				
	*β*_2_	-0.060	0.075	0.420
	*β*_3_	-0.093	0.088	0.295
Insomnia				
	*β*_2_	-0.359	0.080	<.001^∗∗∗^
	*β*_3_	0.046	0.083	0.627
Appetite loss				
	*β*_2_	-0.354	0.072	<.001^∗∗∗^
	*β*_3_	-0.026	0.080	0.747
Constipation				
	*β*_2_	-0.325	0.077	<.001^∗∗∗^
	*β*_3_	0.003	0.083	0.974
Diarrhea				
	*β*_1_	0.739	0.272	0.007 ^∗∗^
	*β*_2_	0.018	0.067	0.792
	*β*_3_	-0.026	0.076	0.786
Financial difficulties				
	*β*_2_	-0.522	0.282	0.066
	*β*_3_	0.302	0.208	0.146

All HRQoL dimensions of the EORTC QLQ-C30 are considered

*P-value <.05; **P-value <.01; ***P-value <.001

HRQoL also changed over time for several of the other dimensions (emotional functioning, pain, insomnia, constipation and appetite loss) resulting in a significant improvement in terms of HRQoL perception. Only the pain dimension showed a significantly different evolution between the two arms (*P-* *value*=0.04). Patients receiving Folfirinox had a perception of pain which decreased significantly more over time than that of the patients receiving Gemcitabine.

**Fig. 2:**
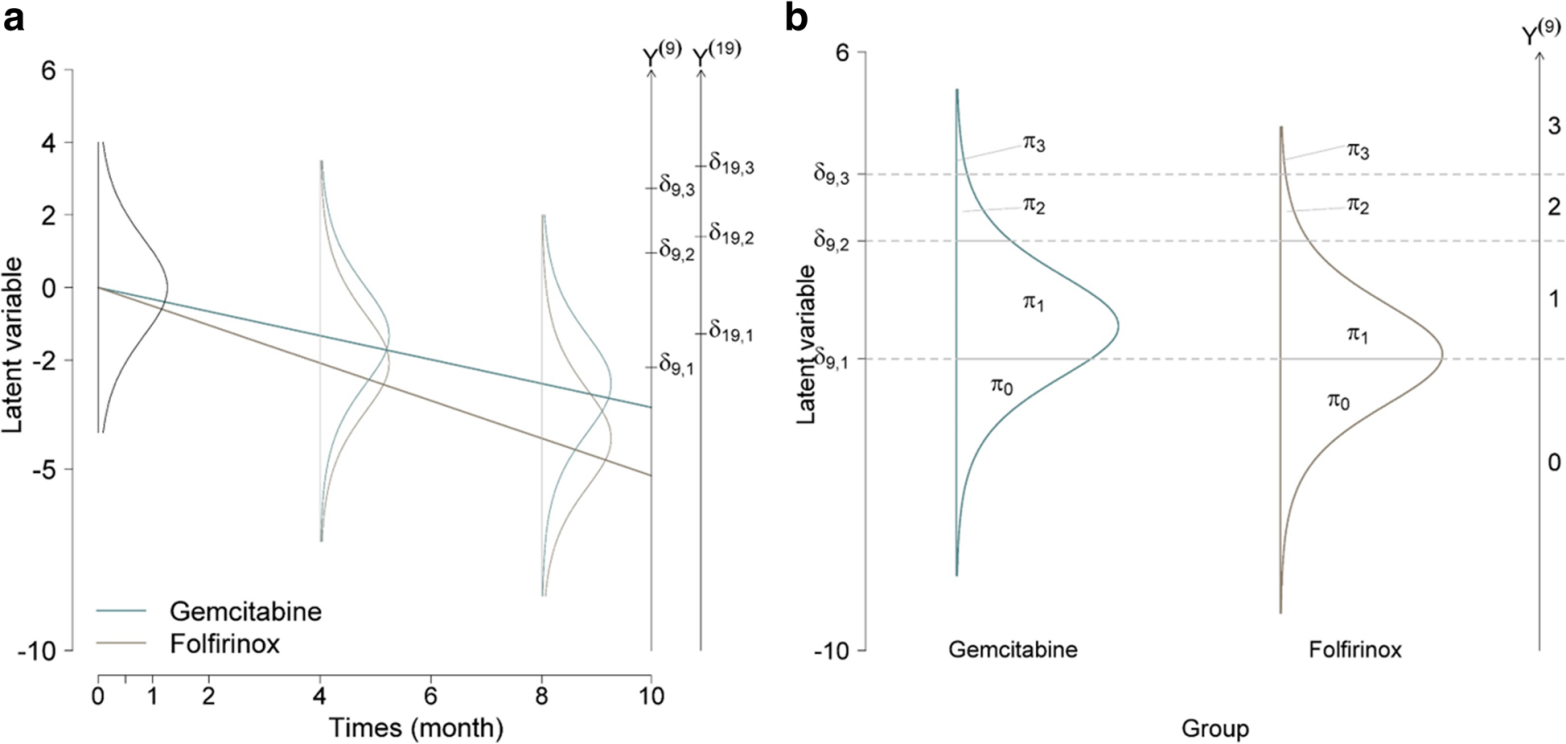
Interpretation of the *(cumulative,logistic,* *Z*_1_,*U*_2_ *)* model through its underlying latent variable concerning the pain symptom (including the items 9 and 19). The estimated difficulty parameter for the item 9 are *δ*_9,1_=−2.1, *δ*_9,2_=1 and *δ*_9,3_=2.75, and for the item 19 : *δ*_19,1_=−1.28, *δ*_19,2_=1.40 and *δ*_19,3_=3.34. **a** the different HRQoL evolution of the latent variable and response variable (*Y* ^(*j*)^)_*j*=9,19_ between the two groups. **b** the different proportions (*π*_*m*_) of different response categories of *Y* ^(9)^ between the two groups four months after the Baseline (*t*_4_)

One of the many advantages of the cumulative models regards the interpretation of results. The constraints on the item parameter in these models allows for interpretation through the latent response variable (i.e. comparing the proportion of patients that selected a response category for one specific item over time or between different groups during a fixed time. Figure 2 shows HRQoL evolution concerning the probability of a response either over time (Fig. 2 a) or between groups (Fig. 2 b). It specifically shows the first item of the pain symptoms from the clinical trial previously described. The probability (*π*_*m*_) for a patient to respond category *m* corresponds to the area under the curve delimited by the horizontal lines. Figure 2 a shows for both groups the probability of choosing categories 2 or 3 decreased over time, whilst the probability of choosing category 0 increased. At baseline, the response proportions for categories 0, 1, 2 and 3 were respectively, *π*_0_=0.10, *π*_1_=0.62, *π*_2_=0.22 and *π*_3_=0.06 for each group. The evolution of the proportion of patients selecting each category showed a decrease in the level of pain between baseline and at the 4 month visit, and finally, a decrease in the latent trait over time. Likewise, Fig. 2 b shows the different response proportions between the two groups at 4 months. For control group, the proportions were *π*_0_=0.29, *π*_1_=0.61, *π*_2_=0.08 and *π*_3_=0.02 for categories 0, 1, 2 and 3, respectively. For experimental group, they were *π*_0_=0.47, *π*_1_=0.48, *π*_2_=0.04 and *π*_3_=0.01. The probability of responding to category 3 was the lowest whatever the group, but was even less likely for patients in the experimental group than those in the control group. On the contrary, patients in the experimental group were more likely to select category 0, than those in the control group. The observed gap corresponds to the difference between the two linear predictors associated with each group only 4 months after the baseline. One of the benefits of this illustration regards the clinical interpretation of the results. The IRT models thus offer a complete analysis: the general analysis of a HRQoL dimension and the specific analysis for each item [8].

## Discussion

We have explored the different suitable mixed models used for the longitudinal analysis of HRQoL in oncology. Using data originating from questionnaires employing Likert scales, we focused on regression models for ordinal data. These models have been specified in terms of linear predictor parameterization, the ratio of probabilities and the CdF [13]. In oncology, analysis is performed on multiple-item measurements associated with one HRQoL dimension [4], the specific IRT parameterization of the linear predictor is thus used. The item parameters allow us to distinguish the outcomes from different items which measure a unique unidimensional latent variable. This latent variable was decomposed linearly to take into account the different covariates in the fixed part of the model and to incorporate subject-specific random effects. Analysis using IRT models is richer than analysis using classical methods, because IRT models are based on raw data [6]. An analysis can be performed on one specific item through the item parameters or on the whole HRQoL dimension [8]. Indeed, these models take into consideration all available information from the data, it is why the use of this kind of model is becoming more and more common [6].

Concerning the decision as to which of the model families to use, the cumulative and adjacent models are preferred. Due to the ratio of probabilities which characterize these models and a symmetric CdF, the practical properties of the invariant under the reverse permutation is an important factor to remember when interpreting the results. The cumulative models also assume an underlying continuous latent response variable [18, 29]. This allows for a better interpretation and illustration of the results. However, the adjacent models have the advantage of not having any constraints in estimation process. These models are thus preferred when the regression and analysis concern the item part of the linear predictor, given non-proportional design. Finally, the choice of the CdF essentially depends on the observed data and properties which interest the users. These IRT models are reversible only if the CdF is symmetrical. Therefore, the use of a commonly symmetrical CdF is preferred (the logistic and the Gaussian distributions).

The simulation study showed that the capacity of the IRT models to detect the random effect was better than that of the classically used LMM. This result was expected, as the LMM is based on the study of a summary variable with less information. Moreover, the capacity of the LMM was not homogeneous following the different scenarios, and it can then influence the ordinal characteristics of the raw data. Concerning the IRT models, the ones that did not generate the dataset seemed more sensitive to the random slope than the IRT model used to generate the dataset. Indeed, in some cases, the model tended to detect the random slope when it did not exist. Then, in the case where one of the two models detects the random slope, it seems that the use of the model not detecting the effect as it is would be is the most appropriate choice, when the decision as to which model to use is data-driven.

When we applied the *(cumulative,logistic,* *Z*_1_,*U*_2_ *)* model to the clinical trial dataset outlined above, it was found that although Folfirinox is known to be more toxic than Gemcitabine, and caused significantly more diarrhea during its administration, the pain perception with Folfirinox decreased significantly more over time compared to that for the patients receiving Gemcitabine. Otherwise, both treatments are equivalent regarding HRQoL evolution over time.

## Conclusions

Research into the statistical analysis used to assess HRQoL is of major importance in enabling clinicians to better evaluate the impact of different treatments on the everyday life of patients and to improve their care. Amongst the models that are used for the longitudinal analysis of HRQoL, we focused on the mixed models from IRT, which are thought to be the most suitable to directly analyze raw data from questionnaires. In this article, the different IRT models for ordinal responses are reviewed using a recent classification of generalized linear models for categorical data. This allowed us to consider a conceptual selection of these models for the different analytical aims, based on theoretical and practical arguments to justify the use of one model over another one. Concerning the longitudinal analysis of HRQoL in cancer clinical trials, the cumulative model from IRT with proportional design and symmetrical CdF produces results that are easier to interpret than those from the adjacent model. Conversely, the adjacent model is more flexible, as there are no parameter constraints, and it seems more suitable than the IRT cumulative model for non-proportional design.

The multidimensional aspect of HRQoL remains to be discussed. Presently in oncology, the different dimensions are analyzed independently of one another, thus resulting in the use of multiple tests, which can be problematic. Moreover, there can be latent relationships present between certain HRQoL dimensions, and a more complete analysis of these relationships may be of interest. One approach that would take into consideration all HRQoL data would be the use of structural equation modeling. This could show the influence of each HRQoL dimension through different factors to explain the global HRQoL, and any potential structural links between the latent variables.

## Additional file

Additional file 1 Annotated SAS codes to fit the adjacent and cumulative models described in simulation section with the *PROC nlmixed*. (PDF 558 Kb)

## Abbreviations

CdF: Cumulative distribution function
EORTC: European organization for research and treatment of cancer
GLM: Generalized linear model(s)
GLMM: Generalized linear mixed model(s)
HRQoL: Health-related quality of life
IRT: Item response theory
LMM: Linear mixed model(s)
QLQ-C30: Quality of Life Questionnaire - Core 30
