# Supplementary material for: Item response models for the longitudinal analysis of health-related quality of life in cancer clinical trials
Source: BMC Med Res Methodol. 2017 Sep 26;17:148. doi: 10.1186/s12874-017-0410-9 (PMC5615461; doi:10.1186/s12874-017-0410-9)
Supplement: Additional file 1 — Annotated SAS codes to fit the adjacent and cumulative models described in simulation section with the PROC nlmixed. (PDF 558 Kb) [file 12874_2017_410_MOESM1_ESM.pdf]

Additional file 1.

Annotated SAS codes to fit the adjacent and cumulative models described in simulation section with the *PROC nlmixed*.

1- Some notations used in SAS codes for a dimension including two items (j=1,2) with four responses categories (m=0,1,2,3)

data = table including all data information (Y, npat, time, index_itj);
deltajm = difficulty parameter associated with the item j and the response category m;
djm = positive auxiliary variable for m=2,3 allowing to respect the constraint on difficulty parameters in cumulative models, such as deltaj2=deltaj1+dj2 and deltaj3=deltaj1+dj2+dj3;
beta = fixed parameter beta;
s0, s1, c01 = the component of the variance matrix (variance of the first, variance of the second and covariance, respectively) for the two subject-specific random effects;
index_it*j* = variable belongs to data (in column) and equal to 1 if the observed response Y is associated with the item j, 0 elsewhere;
theta = latent variable representing the concept defined in equation (2) of the manuscript;
time = covariate associated with the time;
xi0 = intercept random effect;
xi1 = slope random effect;
Fm = definition of the logistic cumulative distribution function for m=1,2,3 (for probit link, use probnorm);
pi = the probability associated with the response Y (c.f. equation (6) for cumulative models and equation (7) for adjacent models);
npat = subject ID column in data.

2- Cumulative model defined in simulation section

(cumulative,logistic,Z_1,U_2) :

```
proc nlmixed data = data tech=NEWRAP ITDETAILS;
parms
%initialization of parameters;
delta11=0,d12=0.5,d13=0.5,
delta21=0,d22=0.5,d23=0.5,
beta1=0, s0=0.5,s1=0.5,c01=0;
%parameter constraints;
bounds d12>0;
bounds d13>0;
bounds d22>0;
bounds d23>0;
%auxiliary steps;
aux1=delta11*index_it1+delta21*index_it2;
aux2=(delta11+d12)*index_it1+(delta21+d22)*index_it2;
aux3=(delta11+d12+d13)*index_it1+(delta21+d22+d23)*index_it2;
theta=beta1*time+xi0+xi1*time;
F1=exp(theta-aux1)/(1+exp(theta-aux1));
F2=exp(theta-aux2)/(1+exp(theta-aux2));
F3=exp(theta-aux3)/(1+exp(theta-aux3));
%model;
if Y=0 then pi=1-F1;
if Y=1 then pi=F1-F2;
if Y=2 then pi=F2-F3;
if Y=3 then pi=F3;
ll=log(pi);
model Y ~ general(ll);
random xi0 xi1 ~ normal([0,0],[s0,c01,s1])subject=npat;
estimate 'delta12' delta11+d12;
estimate 'delta13' delta11+d12+d13;
estimate 'delta22' delta21+d22;
estimate 'delta23' delta21+d22+d23;
TITLE '(cumulative,logistic,eta)';

run;
```

3- Adjacent model defined in simulation section

(adjacent,logistic,Z_1,U_2) :

```
proc nlmixed data = data tech=NEWRAP ITDETAILS;
parms
%initialization of parameters;
delta11=-0,delta12=0,delta13=0,
delta21=0,delta22=0,delta23=0,
time=0,interaction=0,s0=0.5,s1=0.5,c01=0;
%auxiliary steps;
aux1=delta11*index_it1+delta21*index_it2;
aux2=delta12*index_it1+delta22*index_it2;
aux3=delta13*index_it1+delta23*index_it2;
theta=beta1*time+xi0+xi1*time;
F1=exp(theta-aux1)/(1+exp(theta-aux1));
F2=exp(theta-aux2)/(1+exp(theta-aux2));
F3=exp(theta-aux3)/(1+exp(theta-aux3));
R1=F1/(1-F1);
R2=F2/(1-F2);
R3=F3/(1-F3);
denom=1+R1+R1*R2+R1*R2*R3;
%model;
if Y=0 then pi=1/denom;
if Y=1 then pi=R1/denom;
if Y=2 then pi=R1*R2/denom;
if Y=3 then pi=R1*R2*R3/denom;
ll=log(pi);
model Y ~ general(ll);
random xi0 xi1 ~ normal([0,0],[s0,c01,s1])subject=npat;
TITLE '(adjacent,logistic,eta)';
run;
```
